# Indicators and Implementation Guidance to Advance Value-Based HIV Care Through People-Centered Metrics

**DOI:** 10.9745/GHSP-D-23-00220

**Published:** 2024-08-27

**Authors:** Emily Harris, Sameera Ali, Josephine Mungurere-Baker, Atlang Mompe, Chintan Maru, Balkrishna Korgaonkar, Shipra Srihari, Yordanos Molla

**Affiliations:** a U.S. Agency for International Development, Washington, DC, USA.; b Leapfrog To Value, Mumbai, India.; c Leapfrog To Value, Washington, DC, USA.; d Local Health System Sustainability Project, Rockville, MD, USA.

## Abstract

We argue that validating person-centered outcome metrics and integrating them into HIV programs may improve patient’s quality of life and health outcomes by informing the provider-client relationship, promoting integrated service delivery at the program level, and influencing policy and budget allocations at the population level.

## Key Messages

- “People-centered” HIV services recognize individuals’ holistic selves—as people with unique and dynamic needs, preferences, and rights who deserve an individualized service experience tailored to their life journey.
- A validated, streamlined set of metrics can help the HIV community drive toward such a “people-centered” orientation and advance the Joint United Nations Programms on HIV/AIDS’ 2025 target of linking at least 90% of people living with HIV and people at risk to people-centered and context-specific integrated services.
- A framework of people-centered outcomes and 6 corresponding metrics were developed with and for the people they serve to elevate monitoring the degree to which an individual is healthy, comfortable, and able to participate and enjoy life events.
- Successfully integrating these person-centered outcome metrics in an existing HIV program can improve quality of life and health outcomes by encouraging providers and program staff to use the indicator data to improve care provision.
- The HIV community is invited to collaboratively refine and validate these people-centered outcome metrics using the Putting People-Centered Metrics for HIV into Practice toolkit.

## INTRODUCTION

In 2022, the U.S. President’s Emergency Plan for AIDS Relief shifted to a “person-centered” or “people-centered” orientation.^1^ Designed to align with the Joint United Nations Programme on HIV/AIDS (UNAIDS) Global AIDS Strategy’s operating principles, the language change from “client-centered” recognizes individuals’ holistic selves—not only as persons living with HIV in need of diagnosis and treatment but also as people with unique and dynamic needs, preferences, and rights who deserve an individualized service experience tailored to their life journey.

The call for a “people-centered” approach has also been expressed through advocacy in the HIV community for a fourth goal—beyond the original 95-95-95 UNAIDS fast-track targets—to achieve and sustain epidemic control. UNAIDS’ 2025 target of linking at least 90% of people living with HIV and people at risk to people-centered and context-specific integrated services demonstrates the success of this advocacy in elevating people-centered outcomes that have not been previously measured in a standardized way in HIV programs, specifically health-related quality of life.^2^ By drawing inspiration from the value-based care movement, metrics to advance progress toward this 90% goal can be an outcome and an enabler of the UNAIDS’ revised 2025 top-line targets.^3^ By defining the “numerator” of value, HIV programs can apply the metrics to measure and incentivize the outcomes that advance an individual’s quality of life and care experience.

Quality-of-clinical care metrics have evolved with a similar focus on people-centeredness and measuring client experience of care.^4^^,^^5^ Recent emphasis on quality of clinical care extends this concept to integration of services and responsiveness to clients’ needs, in addition to safety, effectiveness, efficiency, equitability, and timeliness of clinical care. Measurement of people-centeredness is based on clients’ reported experience of care with respect to effective communication, which is bidirectional between client and care provider; respect and dignity, which encompasses privacy, nondiscrimination, autonomy, confidentiality, and kindness; and emotional support, which includes social support.^6^ People-centered metrics aim to measure what matters to the person as a whole in and outside of the clinical setting, which is key for improving quality of care as well as for setting health system policy priorities.^7^^,^^8^ People-centered metrics can provide a blueprint for how health services and incentives can be designed to provide high-quality care that is responsive to clients’ needs (i.e., keeping in mind how they affect convenience, access, usability, and motivation for clients and providers).

People-centered metrics can provide a blueprint for how health services and incentives can be designed to provide high-quality care that is responsive to clients’ needs.

People-centered metrics can also motivate improved linkages between clinic and community-based services (e.g., by demonstrating how increased access to wrap-around HIV support services, such as economic livelihood strengthening, can improve clinical outcomes). Initiatives to show the utility of people-centered metrics in HIV programming exist across a range of HIV technical areas.^9^^–^^11^

## DEFINING PEOPLE-CENTERED OUTCOMES AND CORRESPONDING METRICS

A validated, streamlined set of metrics can help the HIV community drive toward such a “people-centered” orientation. To define these metrics, the U.S. Agency for International Development (USAID) and its partners Leapfrog to Value and Data for Implementation used a 3-phase approach. First, a conceptual framework for value-based care and the drivers of people-centered outcomes was developed through a consultative process of reviewing and discussing innovative approaches with organizations that are integrating value-based care in HIV programming. Table 1 summarizes 8 case studies that illustrate the critical building blocks of value-based HIV care, based on a review of 27 innovator organizations and innovations drawn from over 300 identified across 7 databases, secondary research, and expert recommendations.^12^ Second, the framework was used to identify people-centered outcomes and implementation considerations through individual and focus group discussions with 45 subject matter experts (representing 64% female and 36% male members of global and national PLHIV Community Advisory Boards, global and sub-Saharan African HIV and health system experts, and academics and monitoring and evaluation specialists). Finally, the outcomes and considerations were translated into a set of metrics, indicator reference sheets,^2^ and implementation considerations^13^ (Table 2).^5^^,^^8^ Ali et al.^14^ and Leapfrog to Value^12^ provide additional details on the indicator development process. If improvements in these outcomes can be measured and incentivized, programs can realize the ultimate benefit of value-based care: a higher-value care experience at sustainable long-term costs.

**TABLE 1.** Innovative Approaches to Integrating Value-Based Care in HIV Programming

**Building Blocks of Value-Based Care**	**Innovators and Innovations**
Create tools to measure what matters for PLHIV	The International Consortium for Health Outcomes Measurement creates standard metric sets on people-centered outcomes. PROgress Implementation Toolkit integrates patient-reported outcomes (PROs) assessments into routine HIV care.
Amplify PLHIV voices and create agency	Ritshizde conducts community-led clinic monitoring and advocacy.
Address social determinants	Seek-GSP organizes group therapy to address mental health of PLHIV.
Transform provider culture	Beyond Bias addresses provider bias to improve client trust. Wild4Life builds trust in the health system to improve care-seeking behavior.
Align payment models with outcomes	PPO Serve leverages private practices to improve PLHIV care.
Integrate measurement, delivery and payment for a holistic approach to value-based care	OLVG integrates value-based care principles in HIV care across the client journey in high-income settings.

Abbreviation: PLHIV, people living with HIV.

**TABLE 2.** Client Experience of HIV Care and Quality of Life Indicators

**Category**	**Domains**	**Indicator Number and Name**
Care experience	Service level Client-provide interactions (effective bidirectional communication) Dignity of care (including nondiscrimination, autonomy, confidentiality, and kindness)^a^ Privacy User-centered health system (including user voices, ease of use of the system, waiting times, choice of provider)^a^	1. Percentage of surveyed clients who would recommend an HIV health service as a proxy measure of their own care
Quality of life	Symptom control and side effects	2. Percentage of surveyed clients who report minimal HIV-related symptoms
	Mental well-being	3. Percentage of surveyed clients who report experiencing minimal mental or emotional problems in the past 2 weeks
	Social support	4. Percentage of surveyed clients who report feeling socially supported by family and/or friends
	Stigma and discrimination	5. Percentage of surveyed clients who report experiencing minimal discrimination as a result of their HIV status
	Financial burden	6. Percentage of surveyed clients who experience financial hardship associated with their HIV diagnosis and/or their HIV-related treatment and care

^a^ Expanded domains based on World Health Organization^6^ and Kruk et al.^8^

We argue that if we can refine and validate these person-centered outcome metrics and if we can successfully integrate them into an existing HIV program, then care and quality of life and ultimately health outcomes will improve because providers and program staff will use indicator data to improve care provision. Achieving this theory of change will require a significant cultural reorientation and thinking more broadly, within and beyond individual HIV programs. Specifically, indicator data can inform the provider-client relationship, promote integrated service delivery at the program level, and influence policy and budget allocations at the population level. Informed by these indicators, providers can modify care provision by personalizing treatment through early diagnosis of psychosocial factors that can influence adherence to treatment, clinical outcomes, and ultimately quality of life. These shifts are critical to an integrated response to the health needs of PLHIV and for reaching and sustaining the last mile in HIV epidemic control.^13^

The data insights from the people-centered metrics can facilitate this shift in multiple ways. The insights from the indicator assessing mental well-being, for example, can sensitize providers to prioritize their client’s psychosocial needs and encourage programs to design services and referral mechanisms that address those needs. For the indicator assessing financial burden, the insights can alert providers to the ways in which financial insecurity can influence their clients’ HIV care and encourage programs to design financial packages and associated services (i.e., differentiated care models, skills and livelihood development). For the indicator assessing social support, the insights can help identify patients whose social isolation may impact their treatment and promote program’s linkages with supportive services such as community-based support groups. These increased performance feedback loops can help improve provider performance, build trust in the health system, and ultimately improve clinical outcomes by encouraging retention and adherence. These examples demonstrate how data insights from the metrics that matter most to individuals can facilitate success by informing provider, program, and policy-level action.

## CONCLUSION

If successful, people-centered metrics have the potential to shape the trajectory of HIV care by encouraging learning, as the metrics can help HIV providers better understand what matters to clients. The metric’s integration can also drive a research agenda on how meeting these needs can better improve treatment adherence and viral suppression. This can lead to program improvements, by complementing existing program monitoring efforts to collect, interpret, and take action on data insights. These improved performance feedback loops can in turn provide opportunities for designing and integrating incentives, by aligning reward systems (both financial and nonfinancial) with delivering people-centered care. Overall, such continuous, iterative feedback on what matters most to people’s care experience can encourage innovation, by revealing and inspiring opportunities for overcoming persistent care challenges.

If successful, people-centered metrics have the potential to shape the trajectory of HIV care by encouraging learning, as the metrics can help HIV providers better understand what matters to clients.

To realize this potential, programs should first consider piloting these metrics to validate and refine them, ensure that they generate meaningful insights, can be collected efficiently, can be implemented within the operational constraints of HIV care, and are sensitive to client needs. As the UNAIDS’ 2025 target of linking at least 90% of people living with HIV and people at risk to people-centered and context-specific integrated services suggests, additional metrics may be needed to reflect the increasing focus on the client’s care experience, and their ability to access integrated care.^2^ Ultimately, value-based care argues that there is an ultimate cost savings due to improved cost efficiencies in care, but this needs to be balanced with the reality that any new metric has immediate costs to the program. There needs to be an assessment of the availability of resources to implement the person-centered care surveys and considerations of how they will be operationalized without impacting service delivery.^2^

USAID—in partnership with the Local Health System Sustainability Project—supported an exploratory qualitative study in Tete Province, Mozambique, to assess the acceptability and feasibility of both the metrics themselves and the survey tool employed to gather client data. The study was designed to understand the relevance of the metrics, and their potential to generate data that can inform improvement in the management and provision of HIV care. Program staff and clients in health facilities found the indicators acceptable, feasible, and relevant for measuring people-centered metrics in HIV programs.^13^ We invite the HIV community to continue collaboratively assessing, refining, and validating these people-centered outcome metrics, using the Putting People-Centered Metrics for HIV into Practice toolkit.^14^

## Notes

**Peer Reviewed**

**First Published Online:** July 26, 2024.
